# Profiling of the mycobiome and metabolome: a comparative study of benign pulmonary nodules and lung adenocarcinoma

**DOI:** 10.3389/fcimb.2026.1732958

**Published:** 2026-02-23

**Authors:** Weiyi Zhang, Yuan Dong, Fangyan Chen, Fang Wang, Jiahui Li, Chunxi Liu, Tianyi Liu, Li Han, Xiaodong Jia

**Affiliations:** 1 Senior Department of Oncology, Chinese PLA General Hospital, Beijing, China; 2 Department of Pharmacy, Medical Security Center Stationed in the 5th Medical Center Pharmacy Room, Chinese PLA General Hospital, Beijing, China; 3 Department for Disinfection and Infection Control, Chinese PLA Center for Disease Control and Prevention, Beijing, China; 4 Chinese PLA Medical School, Beijing, China; 5 Southern Medical University, Guangzhou, China

**Keywords:** association network, benign pulmonary nodules, diagnostic model, gut fungi, lung adenocarcinoma, metabolite

## Abstract

**Introduction:**

Lung adenocarcinoma (LUAD), the most common subtype of non-small cell lung cancer, is a form of malignant pulmonary nodule that requires clinical differentiation from benign pulmonary nodules (BPN). The mechanisms underlying the development of LUAD are complex, and effective non-invasive methods for differentiating BPN from LUAD are lacking. This study aimed not only to distinguish BPN from LUAD using gut fungi and serum metabolites, but also to establish an integrated network of gut fungi–metabolite–cytokine interactions.

**Methods:**

Fecal and serum samples from individuals with BPN and patients with LUAD were subjected to internal transcribed spacer sequencing, ultra-performance liquid chromatography–tandem mass spectrometry, and multiplex Luminex assays to quantify gut fungi, metabolites, and cytokines, respectively.

**Results:**

A significant difference in gut fungal communities was observed between the BPN and LUAD groups. Multiple genera and species were more abundant in LUAD than in BPN. Docosapentaenoic acid n-6 (DPAn-6), indole-3-propionic acid (IPA), and interferon-γ-induced protein 10 (IP-10) were significantly elevated in the LUAD group. The integrated model established using a combination of gut fungi and metabolites demonstrated excellent performance in distinguishing BPN from LUAD. A network of interactions was established among differentially abundant gut fungi, serum metabolites, and cytokines.

**Conclusion:**

Our study identifies a novel panel of fungal and metabolite biomarkers for differentiating between BPN and LUAD, and constructs a multi-omics network that provides new insights into investigating the mechanistic role of gut mycobiota dysbiosis in LUAD.

## 1 Introduction

Cancer is a big challenge to whole words. The field of health sciences and biotechnology play significant role in the diagnosis and management of diverse types of cancer (Siegel et al., 2025; Sadybekov and Katritch, 2023; Khan et al., 2019, 2025). Various tools of bioinformatics help in the development of novel drug and explore the potential mechanism of carcinogenesis of various types of cancer such as lung cancer, liver cancer, colon cancer, CRC, cervical cancer, prostate cancer etc (Wang et al., 2021; Khan et al., 2024, 2017, 2016, 2016). Lung cancer is the leading cause of cancer-related deaths worldwide. According to the histological type, lung cancer is divided into non-small cell lung cancer (NSCLC) and small cell lung cancer (Zhou et al., 2022). Adenocarcinoma is a frequently diagnosed subtype of NSCLC, accounting for approximately 40–50% of cases (Lian et al., 2022). The incidence of lung adenocarcinoma (LUAD) is increasing annually in both developed and developing countries (Zhang et al., 2024a); therefore, its diagnosis is particularly important for early intervention and treatment. Currently, pathological and imaging studies are mainly used to clinically distinguish between benign and malignant tumors; however, these tests have limitations, including being time-consuming, costly, and invasive, as well as involving radiation risk (Imran et al., 2024). Diagnostic models established using diffusion-weighted imaging and inflammation-related biological markers can effectively distinguish benign and malignant nodules or masses; however, they are limited by image quality, scanning time, and lack of specificity (Dang et al., 2023; Zhang et al., 2024b). Distinguishing between benign and malignant nodules is challenging, and existing knowledge remains insufficient, necessitating further exploration from multiple perspectives.

The intestine contains various fungi (Cao et al., 2021). Fungi exist as a minor component of the microbiota and are often ignored in research owing to their low abundance (Ma et al., 2022). Typical fungal cells are more than 100-times larger than bacterial cells (Underhill and Iliev, 2014). However, the importance of fungi is often underestimated. Gut fungal dysbiosis is an important factor in the development of several tumors, including colorectal (Wang et al., 2024a), prostate (Liu et al., 2014), and pancreatic (Li et al., 2023) cancers. A random forest model established using gut fungi has shown excellent performance in predicting responses to immune checkpoint inhibitors (Huang et al., 2023). Our team previously used gut fungi to establish a well-performing diagnostic model between healthy individuals and patients with LUAD (Liu et al., 2023). However, the gut fungal characteristics that differentiate benign nodules from LUAD warrant further investigation and may provide novel biomarkers for differential diagnosis. Furthermore, alterations in the gut microbiota can influence metabolic changes. Metabolites play crucial roles in tumor development; for instance, butyrate directly enhances the antitumor CD8+ T cell response (He et al., 2021), whereas trans-3-indoleacrylic acid facilitates colorectal carcinogenesis (Cui et al., 2024). Nevertheless, the association between gut fungi and their metabolites in lung cancer remains unclear.

In this study, we investigated the mycobiome, metabolome, and cytokines of individuals, aiming to identify new potential biomarkers for differentiating between benign pulmonary nodules (BPN) and malignant tumors, and to establish a multi-omics network for exploring the relationships among gut fungi, serum metabolites, and cytokines in patients with lung cancer.

## 2 Materials and methods

### 2.1 Study population

Seventy (70) fecal and serum samples were prospectively collected from the General Hospital of the Chinese People’s Liberation Army. Participants were excluded if they: 1) had received antibiotic or probiotic treatment within 1 month before the study; 2) had experienced a lung infection within 1 month; 3) had other types of lung cancer or other cancers; or 4) had received long-term immunosuppressive drug treatment. The study population was divided into two groups: BPN (n = 20) and LUAD (n = 50). Detailed information on the participants, including age, body mass index, and smoking history, was recorded. The clinical characteristics are shown in **Supplementary Table S1**. All procedures involving human participants were performed in accordance with the ethical standards of the institutional and/or national research committee and the Declaration of Helsinki. The study protocol was approved by the Ethics Committee of the General Hospital of Chinese People’s Liberation Army (S2022-407–01). All participants provided informed consent.

### 2.2 Internal transcribed spacer sequencing and data processing

DNA was extracted using the HiPure Fecal DNA Kit (Magen, Guangzhou, China) according to the manufacturer’s instructions. DNA concentration and purity were determined using a NanoDrop 2000 spectrophotometer (Thermo Fisher Scientific, Waltham, MA, USA). Fungal internal transcribed spacer (ITS) 2 was amplified using the sequence-specific primers ITS3_KYO2 (5’-GATGAAGAACGYAGYRAA-3’) and ITS4 (5’-TCCTCCGCTTATTGATATGC-3’). PCR amplification of genomic DNA was conducted in a 20 μL reaction volume with the following cycling parameters: initial denaturation at 95°C for 5 min; 33 cycles of denaturation at 95°C for 1 min, annealing at 60°C for 1 min, and extension at 56°C for 1 min; and a final extension at 72°C for 7 min. Amplicons were separated on 2% agarose gels and purified using an AxyPrep DNA Gel Extraction Kit (Axygen Biosciences, Tewksbury, MA, USA). After purification, DNA concentration was measured using a StepOnePlus Real-Time PCR System (Thermo Fisher Scientific) before pooling into libraries. A second round of PCR was then performed in a 50 μL reaction mixture under the following conditions: 95°C for 5 min; 12 cycles of 95°C for 1 min, 60°C for 1 min, and 72°C for 1 min; followed by a final extension at 72°C for 7 min. Subsequently, the polymerase chain reaction products were used for library construction on an Illumina NovaSeq 6000 platform (Illumina, San Diego, CA, USA) and sequenced using a MiSeq PE250 sequencer.

The ACE, Shannon, and Simpson indices were calculated using QIIME v.1.9.1. Wilcoxon rank-sum tests were used to compare alpha diversity indices between groups using the R vegan package v.2.5.3. Principal coordinates analysis (PCoA) based on the Bray–Curtis distance and Adonis tests were performed using the R vegan package v.2.5.3. Venn diagrams were generated to identify unique and shared families, genera, and species between groups. Linear discriminant analysis effect size (LEfSe) v.1.0 was used to identify biomarkers for each group. Circos plots were generated using the OmicStudio online tool (https://www.omicstudio.cn/tool).

### 2.3 Metabolomic analysis of serum samples

Metabolites were quantified using ultra-performance liquid chromatography–tandem mass spectrometry (ACQUITY UPLC-Xevo TQ-S; Waters Corp., Milford, MA, USA) according to the manufacturer’s instructions. A rigorous quality control system was used to ensure the reliability of the results. Raw data were processed using TMBQ v.1.0 (Metabo-Profile, Shanghai, China), including peak integration and quantification. Statistical analyses, including principal component analysis, orthogonal partial least squares discriminant analysis (OPLS-DA), and univariate and pathway analyses, were performed using a self-developed iMAP platform v.1.0 (Metabo-Profile).

### 2.4 Cytokine analysis in serum samples

The levels of 30 cytokines were assessed by multiplex analysis using a Luminex 200 analyzer with a Procarta Plex Immunoassay kit (Thermo Fisher Scientific), according to the manufacturer’s instructions.

### 2.5 Statistical analysis

In the demographic analysis conducted using the R software (version 4.3.1), normally distributed continuous variables were expressed as the mean ± standard deviation (SD), and categorical variables were expressed as numbers (percentages). Intergroup comparisons of cytokines were also performed using R. Normally distributed continuous variables were compared using t-tests, whereas non-normally distributed variables were analyzed using the Wilcoxon test. A two-sided chi-square test or Fisher’s exact test was used to compare categorical variables between the two groups. In this study, 11 fungal species and 10 differential metabolites were selected as feature variables, and group status was used as the dependent variable. Using SPSS v.26.0, a diagnostic model was established to differentiate LUAD from BPN. The discriminatory power of fungi and metabolites in diagnosing BPN and LUAD was evaluated using receiver operating characteristic (ROC) curves and the area under the curve (AUC).

The Youden index was calculated as follows:

(1) Youden index=sensitivity+specificity−1

Fu et al., 2021.

Spearman correlations among key gut fungi, differential metabolites, and cytokines were calculated, and networks were visualized using Cytoscape v.3.10.1. Statistical significance was defined as a two-tailed *P*-value< 0.05.

## 3 Results

### 3.1 Gut fungal signatures of BPN and LUAD

**Figure 1:**
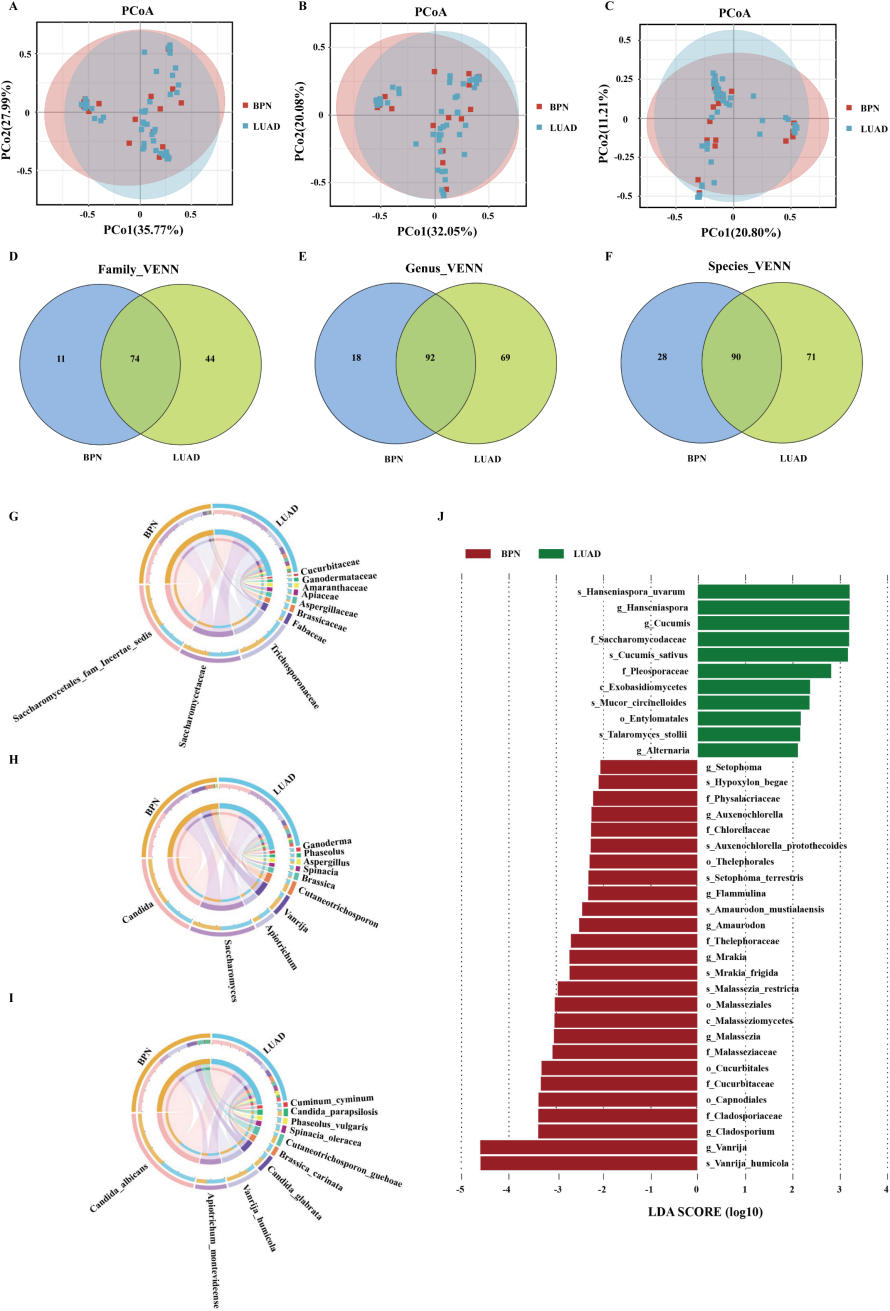
Characteristics of gut microbiota. Significant differences of principal coordinates of the gut microbiota between the benign pulmonary nodule (BPN; red dots) and lung adenocarcinoma (LUAD; blue dots) groups at the family **(A)**, genus **(B)**, and species **(C)** levels. Venn diagrams showing the shared and unique families **(D)**, genera **(E)**, and species **(F)** between the BPN and LUAD groups. Circos plots showing the differences at the family **(G)**, genus **(H)**, and species **(I)** levels between the BPN and LUAD groups. **(J)** Histogram of linear discriminant analysis (LDA) scores representing the effect size and ranking of gut microbiota, in which LDA > 2. Columns in red and green correspond to BPN and LUAD, respectively.

The gut fungal data were generated by ITS2 sequencing. Alpha diversity indices, including the ACE, Shannon, and Simpson indices, were used to assess the sample diversity and richness. No significant differences were observed between the LUAD and BPN groups (**Supplementary Figure S1**). However, significant differences were observed in the gut fungal communities between the two groups based on PCoA at the family, genus, and species levels (*P* = 0.021, 0.005, and< 0.001, respectively; **Figures 1A–C**). Venn diagrams showed that the LUAD group included more fungi at the family, genus, and species levels than the BPN group (**Figures 1D–F**); the two groups shared 74 families, 92 genera, and 90 species.

The main fungal compositions of the BPN and LUAD groups are shown in Circos diagrams (**Figures 1G–I**). At the family level, the BPN and LUAD samples mainly comprised Saccharomycetales fam. Incertae sedis, Saccharomycetaceae, and Trichosporonaceae. At the genus level, the two groups mainly comprised *Candida*, *Saccharomyces*, *Apiotrichum*, *Vanrija*, and *Cutaneotrichosporon*. At the species level, the two groups mainly comprised *Candida albicans*, *Apiotrichum montevideense*, *Vanrija humicola*, *Candida glabrata*, *Brassica carinata*, and *Cutaneotrichosporon guehoae*. LEfSe revealed 11 fungal genera and 11 species that differed between the BPN and LUAD groups (**Figure 1J**). Four species, *Hanseniaspora uvarum*, *Cucumis sativus*, *Mucor circinelloides*, and *Talaromyces stollii*, were enriched in the LUAD group compared with the BPN group.

### 3.2 Altered serum metabolite profiles in BPN and LUAD

**Figure 2:**
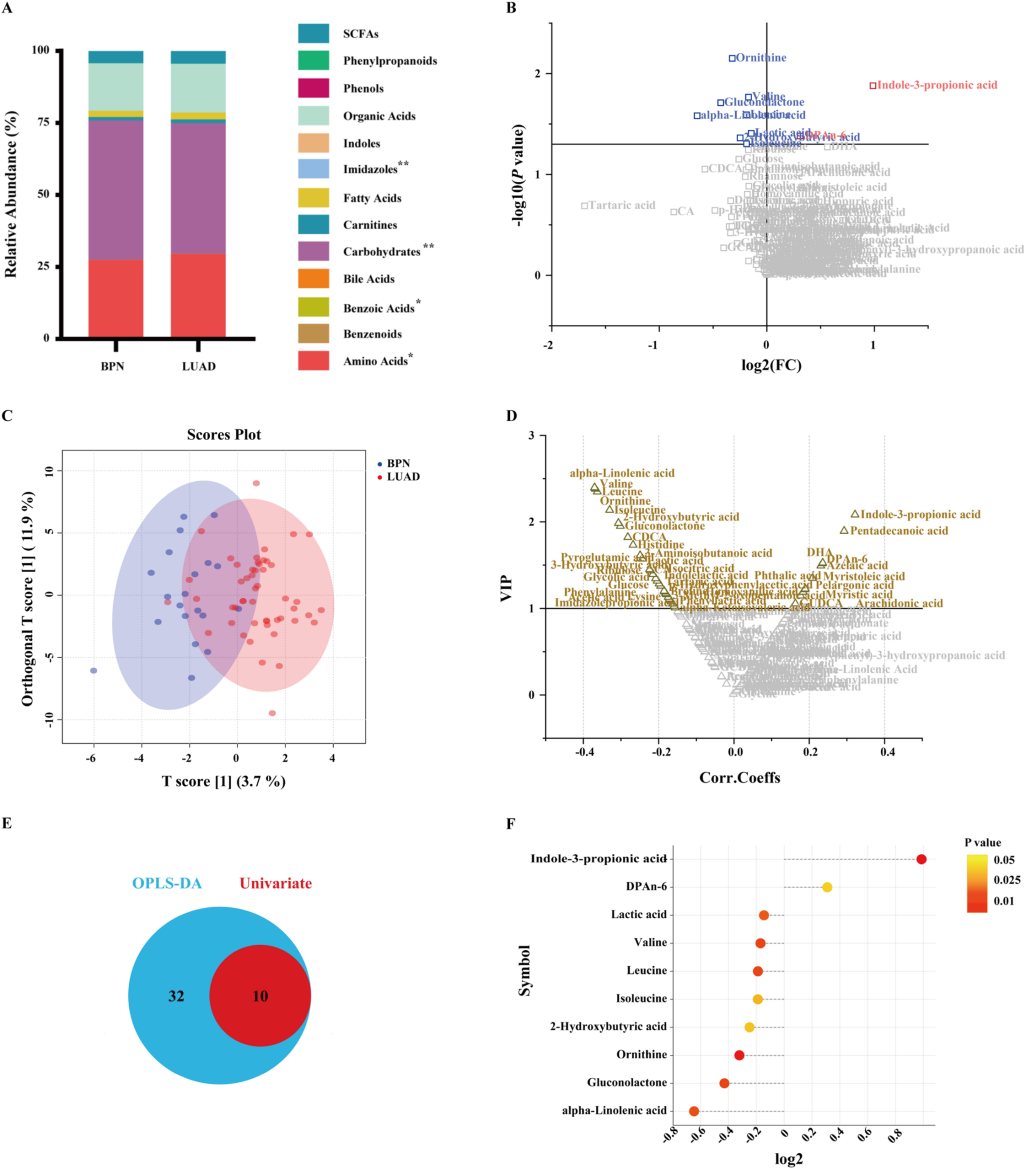
Characteristics of serum metabolites. **(A)** Bar chart representing the relative abundance of serum metabolites in the BPN and LUAD groups (*P< 0.05, **P< 0.01). Volcano plots illustrating differential metabolites selected by univariate **(B)** and multivariate **(D)** analyses. Metabolites in the upper right and left quadrants indicate those upregulated and downregulated, respectively, in the LUAD group, compared with the BPN group. Grey points denote metabolites that did not meet the established threshold criteria. **(C)** Orthogonal partial least squares discriminant analysis (OPLS-DA) score plots showing the separation between patients with LUAD (red) and individuals with BPN (blue). **(E)** Venn diagram showing unique and shared metabolites identified using OPLS-DA and univariate analysis. **(F)** Differences in metabolites between the BPN and LUAD groups, with positive values indicating relatively high metabolite levels in the LUAD group.

Targeted metabolomic analysis of 201 microbial-related and host-specific metabolites detected 127 metabolites (**Supplementary Table S2**). The metabolites were classified into 13 categories: short-chain fatty acids, phenylpropanoids, phenols, organic acids, indoles, imidazoles, fatty acids, carnitines, carbohydrates, bile acids, benzoic acids, benzenoids, and amino acids (**Figure 2A**). In the LUAD group, amino acids, benzoic acids, and imidazoles accounted for a relatively large proportion (*P* = 0.046, 0.043, and 0.007, respectively), whereas carbohydrates accounted for a relatively small proportion (*P* = 0.002).

Univariate analysis identified 10 differential metabolites between the two groups (*P* < 0.05; **Figure 2B**; **Supplementary Table S3**). OPLS-DA, a supervised multivariate method, revealed significant differences in metabolite profiles between the paired groups (**Figures 2C, D**). Forty-two metabolites were selected based on the criteria of variable importance in projection (VIP) > 1 and |*P*(correlation)|< 0.5 (**Supplementary Table S4**). Furthermore, 10 shared metabolites were identified from the results of univariate and multivariate analyses, which were mainly involved in valine, leucine, and isoleucine biosynthesis (**Figure 2E**; **Supplementary Figure S2B**). The VIP scores of the 10 metabolites are shown in **Supplementary Figure S2A**; the top three were ornithine, indole-3-propionic acid (IPA), and valine. In the LUAD group, the levels of IPA and docosapentaenoic acid n-6 (DPAn-6) were relatively high, whereas the levels of eight metabolites, such as ornithine, isoleucine, and valine, were relatively low (**Figure 2F**).

### 3.3 Diagnostic models based on gut fungi and their metabolites

**Figure 3:**
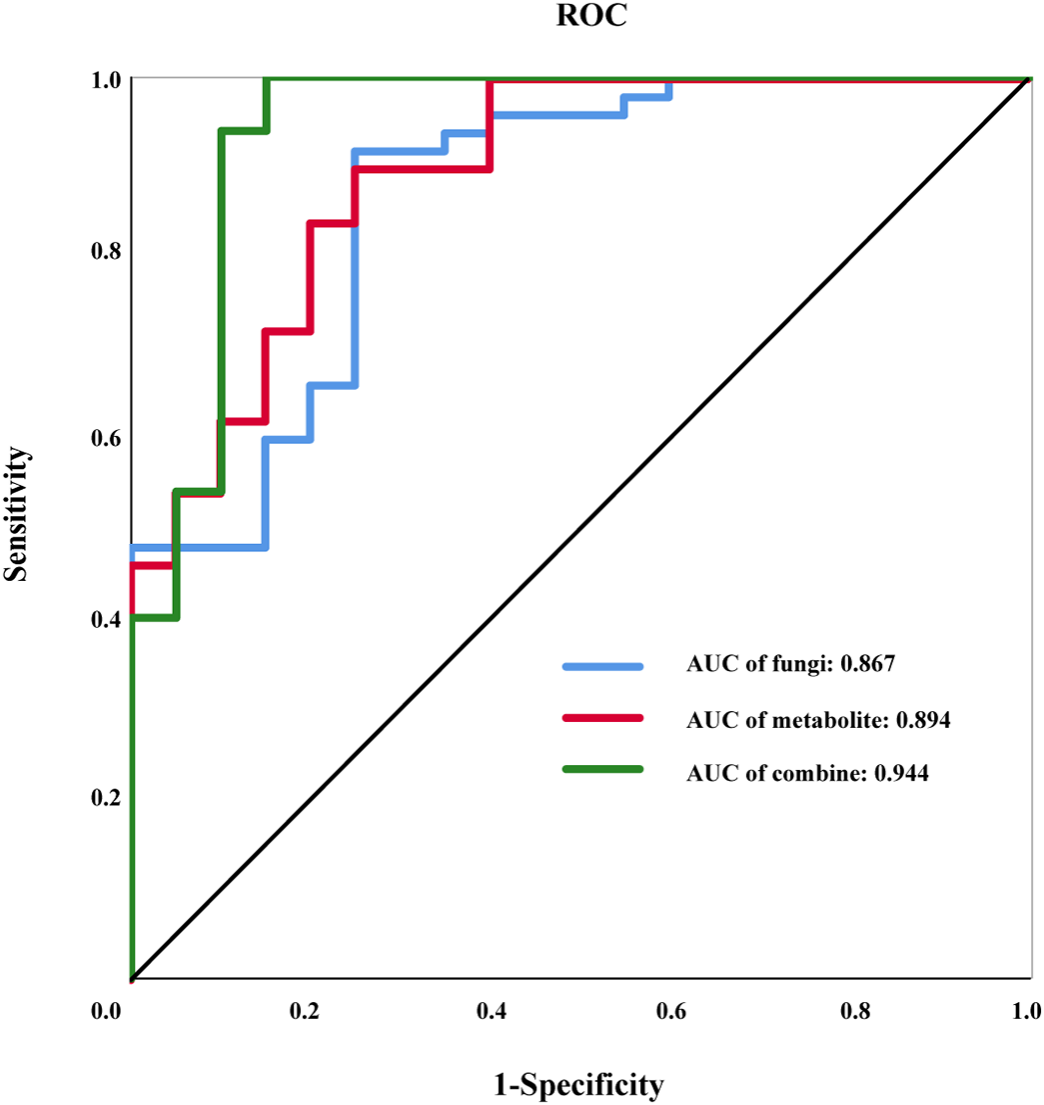
Results of receiver operating characteristic (ROC) analysis. The ROC curves of fungi, metabolites, and their combinations.

We selected 11 fungal species from the LEfSe analysis, including *H. uvarum*, *C. sativus*, *M. circinelloides*, *T. stollii*, *Hypoxylon begae*, *Auxenochlorella protothecoides*, *Setophoma terrestris*, *Amaurodon mustialaensis*, *Mrakia frigida*, *Malassezia restricta*, and *Vanrija humicola* (**Figure 1J**), and 10 differential metabolites (**Figure 2F**) as feature variables, and established an ROC model through binary logistic regression analysis to distinguish LUAD from BPN. **Figure 3** presents the ROC curve results. The AUC of the fungus model was 0.867 [95% confidence interval (CI): 0.771–0.963; *P* < 0.001]. The Youden index (**Equation 1**) was used to determine the highest sensitivity and specificity. The corresponding cutoff value for the diagnosis of the fungal model was 0.67, and the sensitivity and specificity were 92% and 75%, respectively. The AUC of the metabolite model was 0.894 [95% CI: 0.811–0.977; *P* < 0.001]. The corresponding cutoff value for the diagnosis of differential metabolites was 0.566, and the sensitivity and specificity were 90% and 75%, respectively. The AUC of the integrated model with fungal species and metabolites was 0.944 [95% CI: 0.872–1; *P* < 0.001]. The corresponding cutoff value for the integrated diagnosis was 0.67, and the sensitivity and specificity were 92% and 75%, respectively.

### 3.4 Gut fungi, metabolites, and cytokines form an association network

**Figure 4:**
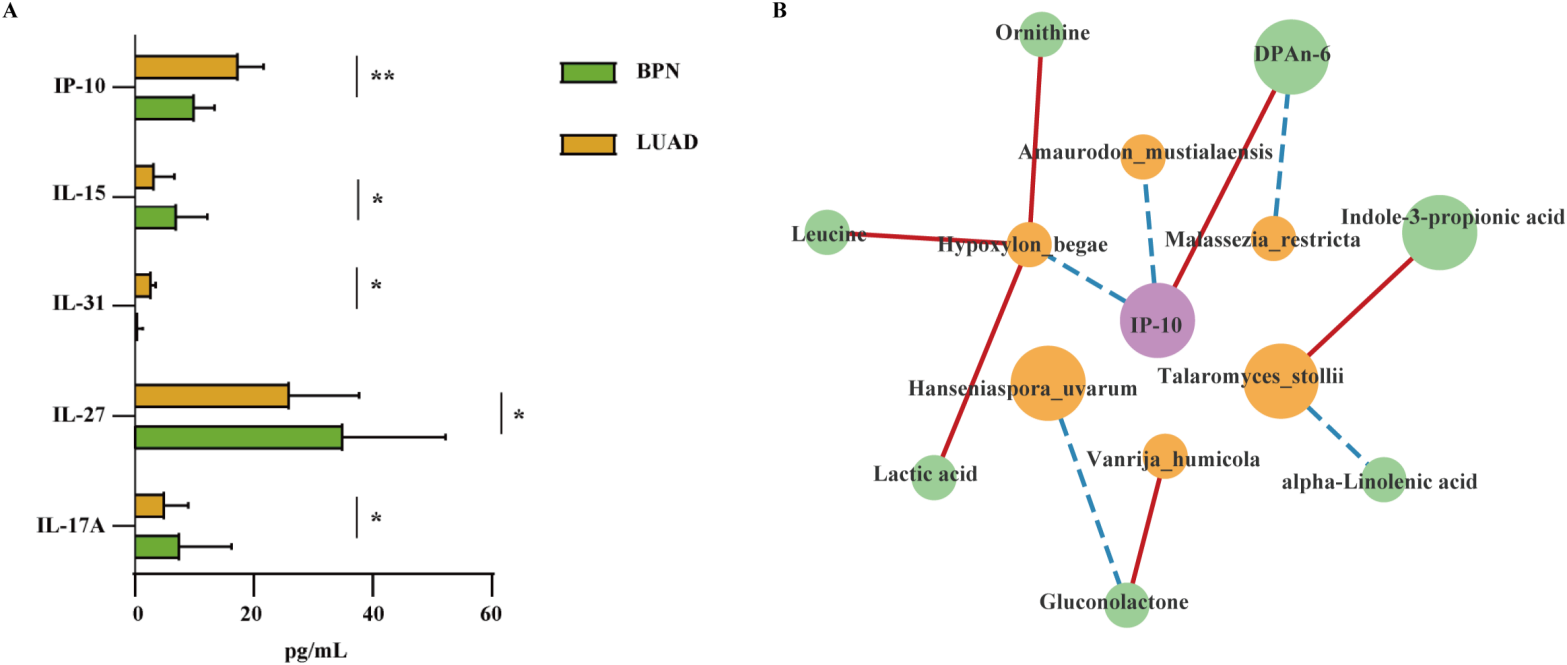
Establishing the gut fungi–metabolite–cytokine interrelationships. **(A)** Significant differences in the levels of cytokines interferon-γ-induced protein 10 (IP-10), interleukin (IL)-15, IL-31, IL-27, and IL-17A between the LUAD and BPN groups (*P < 0.05). **(B)** Interaction network among gut fungi at the species level, differential metabolites, and cytokines in LUAD. The blue dashed and red solid lines indicate negative and positive correlations, respectively. Yellow, fungal species; green, differential metabolites; purple, cytokines. Large circles indicate a higher abundance in the LUAD group.

Abnormal expression of cytokines occurs in LUAD (Xu et al., 2022). In the present study, the levels of interferon-γ (IFN-γ)-induced protein 10 (IP-10) and interleukin (IL)-31 were higher (*P* = 0.0041 and 0.0488, respectively), whereas the levels of IL-15, IL-27, and IL-17 were lower (*P* = 0.0248, 0.0462, and 0.0226, respectively) in the LUAD group than in the BPN group (**Figure 4A**, **Supplementary Table S5**). The interactions among microbiota, metabolites, and cytokines play significant roles in cancer. Therefore, a correlation network comprising 11 fungal species, 10 metabolites, and 5 cytokines was established (**Figure 4B**). The abundance of *H. begae* was positively correlated with lactic acid, leucine, and ornithine levels (*P* = 0.015, 0.021, and 0.018, respectively). The abundance of *T. stollii* was positively correlated with IPA levels (*P* = 0.011) and negatively correlated with alpha-linolenic acid levels (*P* = 0.01). Gluconolactone levels were positively correlated with the abundance of *V. humicola* (*P* = 0.034) and negatively correlated with the abundance of *H. uvarum* (*P* = 0.015). High IP-10 levels were associated with low abundances of *H. begae* and *A. mustialaensis* (*P* = 0.015 and 0.037, respectively).

## 4 Discussion

Healthy individuals and patients with LUAD show significant differences in the composition of the gut microbiota (Chen et al., 2024). Most studies have focused on the bacteriome, whereas studies on gut fungi are limited. The differences in mycobiomes of individuals with BPN and patients with LUAD remain unclear. The gold standard for differentiating BPN from LUAD is pathological diagnosis, which is an invasive procedure with inherent risks.

This study, for the first time to our knowledge, established a differential diagnostic model for LUAD by combining gut fungi and metabolites, demonstrating excellent performance and indicating promising clinical applications. Several studies have established diagnostic models for distinguishing lung cancer from benign lung diseases using features such as DNA methylation (Wang et al., 2023) and radiomics data (Piskorski et al., 2025); however, the AUC values reported in these studies are lower than that of our model. Given the limited sample size, we plan to expand the cohort in future studies for further validation.

In the present study, four fungal species were found to be more abundant in LUAD than in BPN. Among them, *H. uvarum* and *T. stollii* were associated with the indicated metabolites. Moreover, *H. uvarum* was the most abundant species in the LUAD group. *H. uvarum*, an apiculate yeast species, helps in wine fermentation; ethyl acetate produced by *H. uvarum* is a potential biocontrol agent, which can prevent mycotoxin contamination (Liu et al., 2022). *H. uvarum* was significantly negatively correlated with carbohydrates (gluconolactone). This is consistent with the findings of a previous study on fruit eroded by fruit flies which indicated that, in samples treated with *H. uvarum*, carbohydrate concentrations significantly decreased over time, probably owing to the production of volatile compounds by *H. uvarum* that are attractive to flies; this strategy can improve the efficacy of attract-and-kill formulations against fruit flies (Bianchi et al., 2020). *T. stollii*, a species of ascomycete fungi, can effectively inhibit the growth of various plant pathogenic fungi (specifically *Fusarium* species) and suppress mycotoxin production by generating active antifungal compounds such as talaromyolides (Vignassa et al., 2024). In the present study, *T. stollii* was negatively correlated with alpha-linolenic acid. Kefir, a probiotic-containing fermented beverage, plays an important role in colorectal cancer. Kefir consumption by patients with colorectal cancer increases fatty acid levels, inhibits the growth of *Talaromyces*, regulates the composition of the intestinal flora, enhances the intestinal barrier integrity, and improves colorectal cancer outcomes (Zeng et al., 2021). *Alternaria*, which is involved in multiple diseases, may be associated with LUAD. *Alternaria* is enriched in the intestines of patients with gastric and esophageal cancers (Zhong et al., 2021; Yuan et al., 2025), which is consistent with the results of our study. In pancreatic ductal adenocarcinoma, *Alternaria* mediates IL-33 secretion from tumors, attracting type 2 immune cells, thereby promoting tumor progression (Alam et al., 2022). In addition, *Alternaria* produces various mycotoxins that may participate in the progression of renal cancer through synergistic effects at low concentrations or antagonistic effects at high concentrations (Claeys et al., 2022). However, the roles of *H. uvarum*, *T. stollii*, and *Alternaria* in LUAD remain largely unexplored and require further investigation. Research on *Hanseniaspora uvarum* and *Talaromyces stollii* in the human body remains limited, necessitating validation with larger sample sizes and in-depth investigation into the mechanisms of *Alternaria* in LUAD.

Among the 10 metabolite biomarkers identified, only IPA and DPAn-6 levels were higher in LUAD. The level of IPA, a deamination product of tryptophan, significantly increased in the saliva of patients with oral cancer (Wei et al., 2011), which is consistent with the results of our study. High doses of IPA result in poor survival rates of larvae (Edzeamey et al., 2025). Moreover, IPA may exert anti-cancer effects (e.g., lung cancer) by inhibiting cell proliferation, inducing apoptosis, and promoting oxidative stress and inflammation in the tumor microenvironment (Guo et al., 2017; Miao et al., 2025). DPAn-6 is a member of the ω-3 polyunsaturated fatty acid family and is primarily synthesized in the liver from arachidonic acid (20:4n-6) and/or linoleic acid (18:2n-6). Its accumulation in the brain is positively associated with behavioral and neurological impairments in rodents (Igarashi et al., 2012). Elevated levels of circulating DPAn-6 are linked to poor cognitive performance in individuals aged 60–70 years from high-income backgrounds (Lin et al., 2025) and may serve as a potential biomarker for a high risk of depression among adults in the US (Wang et al., 2024b). Therefore, DPAn-6 may play an important role in neurological health. Moreover, it exhibits anti-inflammatory activity (Nauroth et al., 2010). In our study, DPAn-6 levels positively correlated with IP-10, although no existing studies have reported a relationship between these two factors. As a chemokine, IP-10 levels were higher in patients with LUAD than in individuals with BPN in our study. Similarly, IP-10 levels have been reported to be significantly higher in the peripheral blood of patients with NSCLC than in that of healthy controls (Guo et al., 2019). Moreover, IP-10 expression in tumor tissues of NSCLC have been found to be significantly higher than that in tumor-adjacent and normal tissues, and the median progression-free survival (mPFS) of patients with IP-10^low^ has been significantly lower than that of patients with IP-10^high^ (Cao et al., 2017). IP-10 is inversely associated with the risk of lung cancer in ever smokers (Luo et al., 2024). Patients with NSCLC and early high-grade pulmonary toxicity after radiotherapy show low levels of IP-10 (Siva et al., 2014). IP-10 is an angiostatic factor, which inhibits NSCLC tumorigenesis and spontaneous metastases (Arenberg et al., 1996). Therefore, IP-10 may suppress LUAD; however, whether DPAn-6 has a similar inhibitory effect remains unclear. The roles of IPA and DPAn-6 in LUAD, as well as the relationship between DPAn-6 and IP-10 require further experimental validation.

In conclusion, this study combined gut fungi and metabolites as integrated biomarkers to construct a comprehensive diagnostic model for discriminating between BPN and LUAD. Furthermore, based on statistical screening results, the interactions among gut fungi, metabolites, and cytokines were further explored, providing new insights into the mechanistic link between gut mycobiota dysbiosis and lung cancer development.

## Data Availability

The original contributions presented in the study are included in the article/**Supplementary Material**. Further inquiries can be directed to the corresponding author.
