# Supplementary material for: Profiling of the mycobiome and metabolome: a comparative study of benign pulmonary nodules and lung adenocarcinoma
Source: Front Cell Infect Microbiol. 2026 Feb 23;16:1732958. doi: 10.3389/fcimb.2026.1732958 (PMC12968269; doi:10.3389/fcimb.2026.1732958)
Supplement: Supplementary file 1 [file DataSheet1.pdf]

Supplementary Material

1 Supplementary Figures and Tables

1.1 Supplementary Figures

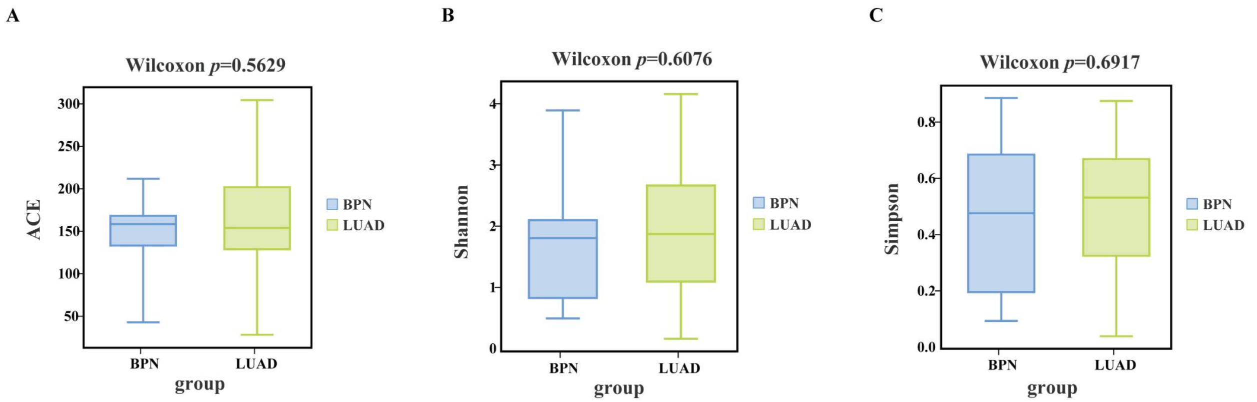

Figure S1. Characteristics of Gut Microbiota Alpha Diversity. Comparison of ACE (A), Shannon (B), and Simpson (C) indices between BPN and LUAD patients. Blue represents BPN, and green represents LUAD.

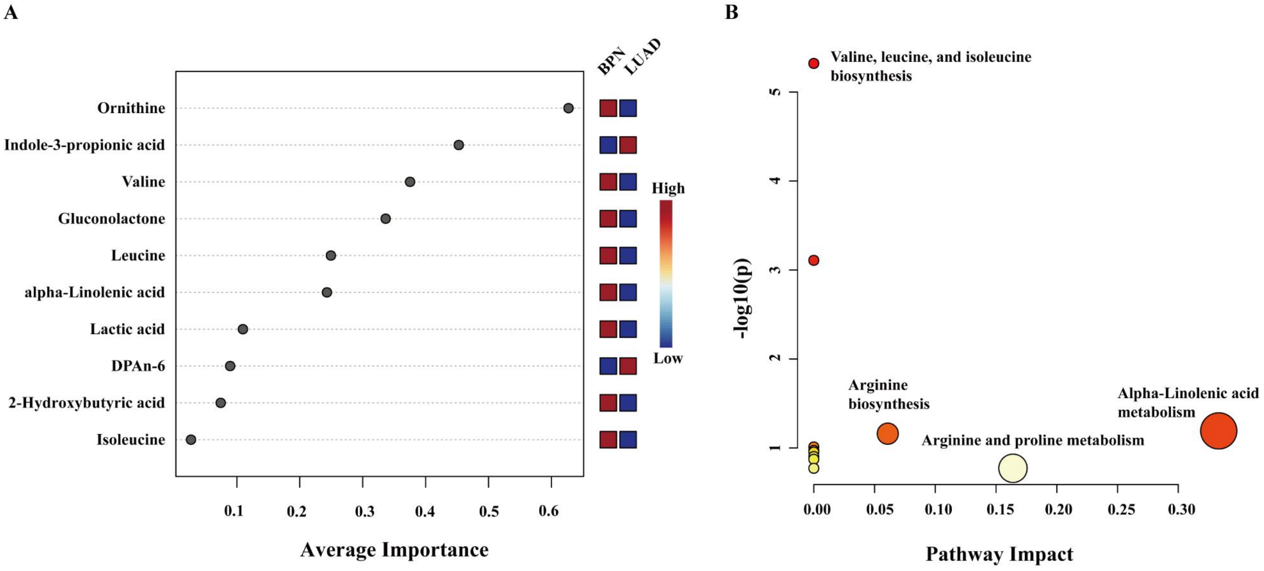

Figure S2. Metabolite VIP Scores and Pathway Analysis.(A) In the VIP score plot, the x-axis represents the average importance, and the y-axis represents the ranking of metabolites. Red represents BPN, and blue represents LUAD. (B) In the KEGG pathway plot of metabolites, the x-axis represents the pathway impact, and the y-axis represents -log10(p).

1.2 Supplementary Tables

Table S1. Clinical Characteristics of the Study Population

**Variable**	**Total (n=70)**	**BPN group (n=20)**	**LUAD group (n=50)**	***P* Value**
Age	55.70±9.86	55.05±9.76	55.94±9.99	0.71
BMI	24.70±3.53	25.10±4.14	24.54±3.29	0.55
Gender				
Male	38 (54.3)	15 (75.0)	23 (46.0)	0.04
Female	32 (45.7)	5 (25.0)	27 (54.0)	
Smoking History				
No	45 (64.3)	11 (55.0)	34 (68.0)	0.31
Yes	25 (35.7)	9 (45.0)	16 (32.0)	
Drinking History				
No	31 (44.3)	13 (65.0)	18 (36.0)	0.063
Yes	39 (55.7)	7 (35.0)	32 (64.0)	

Table S2. List of Quantified Metabolites

Metabolites	HMDB_ID	Kingdom	Super Class	CLASS	SUB CLASS	DIRECT PARENT	PubChem	KEGG
Caproic acid	HMDB0000535	Organic compounds	Lipids and lipid-like molecules	Fatty Acyls	Fatty acids and conjugates	Medium-chain fatty acids	8892	C01585
Ethylmethylacetic acid	HMDB0002176	Organic compounds	Lipids and lipid-like molecules	Fatty Acyls	Fatty acids and conjugates	Methyl-branched fatty acids	8314	C18319
2_Hydroxy_3_methylbutyric acid	HMDB0000407	Organic compounds	Lipids and lipid-like molecules	Fatty Acyls	Fatty acids and conjugates	Hydroxy fatty acids	99823	NA
2_Hydroxybutyric acid	HMDB0000008	Organic compounds	Organic acids and derivatives	Hydroxy acids and derivatives	Alpha hydroxy acids and derivatives	Alpha hydroxy acids and derivatives	440864	C05984
2_Methylhexanoic acid	HMDB0031594	Organic compounds	Lipids and lipid-like molecules	Fatty Acyls	Fatty acids and conjugates	Medium-chain fatty acids	20653	NA
2_Phenylpropionate	HMDB0011743	Organic compounds	Phenylpropanoids and polyketides	Phenylpropanoic acids	NA	Phenylpropanoic acids	10296	NA
3_ 3_Hydroxyphenyl _3_hydroxypropanoic acid	HMDB0002643	Organic compounds	Phenylpropanoids and polyketides	Phenylpropanoic acids	NA	Phenylpropanoic acids	102959	NA
3_Methyladipic acid	HMDB0000555	Organic compounds	Lipids and lipid-like molecules	Fatty Acyls	Fatty acids and conjugates	Medium-chain fatty acids	6999745	NA
3_Aminosalicylic	HMDB000	Organic compo	Benzenoids	Benzene and substituted	Benzoic acids and	Aminosalicylic acids	68443	NA

acid	1972	unds		derivatives	derivatives			
3_Hydroxy butyric acid	HMDB0000357	Organic compounds	Organic acids and derivatives	Hydroxy acids and derivatives	Beta hydroxy acids and derivatives	Beta hydroxy acids and derivatives	92135	C01089
m_Hydroxyhippuric acid	HMDB0006116	Organic compounds	Benzenoids	Benzene and substituted derivatives	Benzoic acids and derivatives	Hippuric acids	450268	NA
3_Methyl_2_oxovaleric acid	HMDB0000491	Organic compounds	Organic acids and derivatives	Keto acids and derivatives	Short-chain keto acids and derivatives	Short-chain keto acids and derivatives	439286	C00671
Acetic acid	HMDB0000042	Organic compounds	Organic acids and derivatives	Carboxylic acids and derivatives	Carboxylic acids	Carboxylic acids	176	C00033
L_Acetylcarnitine	HMDB0000201	Organic compounds	Lipids and lipid-like molecules	Fatty Acyls	Fatty acid esters	Acyl carnitines	7045767	C02571
Acetylglycine	HMDB0000532	Organic compounds	Organic acids and derivatives	Carboxylic acids and derivatives	Amino acids, peptides, and analogues	N-acyl-alpha amino acids	10972	NA
Adrenic acid	HMDB0002226	Organic compounds	Lipids and lipid-like molecules	Fatty Acyls	Fatty acids and conjugates	Very long-chain fatty acids	5497181	C16527
L_Alanine	HMDB0000161	Organic compounds	Organic acids and derivatives	Fatty Acyls	Amino acids, peptides, and analogues	Alanine and derivatives	5950	C00041
alpha_Hydroxyisobutyric acid	HMDB0000729	Organic compounds	Organic acids and derivatives	Hydroxy acids and derivatives	Alpha hydroxy acids and derivatives	Alpha hydroxy acids and derivatives	11671	NA

Alpha_ketoisovaleric acid	HMDB0000019	Organic compounds	Organic acids and derivatives	Keto acids and derivatives	Short-chain keto acids and derivatives	Short-chain keto acids and derivatives	49	C00141
Alpha_Linolenic acid	HMDB0001388	Organic compounds	Lipids and lipid-like molecules	Fatty Acyls	Lineolic acids and derivatives	Lineolic acids and derivatives	5280934	C06427
Arachidonic acid	HMDB0001043	Organic compounds	Lipids and lipid-like molecules	Fatty Acyls	Fatty acids and conjugates	Long-chain fatty acids	444899	C00219
L_Arginine	HMDB0000517	Organic compounds	Organic acids and derivatives	Fatty Acyls	Amino acids, peptides, and analogues	L-alpha-amino acids	6322	C00062
L_Asparagine	HMDB0000168	Organic compounds	Organic acids and derivatives	Carboxylic acids and derivatives	Amino acids, peptides, and analogues	Asparagine and derivatives	6267	C00152
L_Aspartic acid	HMDB0000191	Organic compounds	Organic acids and derivatives	Carboxylic acids and derivatives	Amino acids, peptides, and analogues	Aspartic acid and derivatives	5960	C00049
Azelaic acid	HMDB0000784	Organic compounds	Lipids and lipid-like molecules	Fatty Acyls	Fatty acids and conjugates	Medium-chain fatty acids	2266	C08261
Beta_Ursodeoxycholic acid	HMDB0000686	Organic compounds	Lipids and lipid-like molecules	Steroids and steroid derivatives	Bile acids, alcohols and derivatives	Dihydroxy bile acids, alcohols and derivatives	127601	C17662
Butyric acid	HMDB0000039	Organic compounds	Lipids and lipid-like molecules	Fatty Acyls	Fatty acids and conjugates	Straight chain fatty acids	264	C00246

Cholic acid	HMDB0000619	Organic compounds	Lipids and lipid-like molecules	Steroids and steroid derivatives	Bile acids, alcohols and derivatives	Trihydroxy bile acids, alcohols and derivatives	221493	C00695
Capric acid	HMDB00511	Organic compounds	Lipids and lipid-like molecules	Fatty Acyls	Fatty acids and conjugates	Medium-chain fatty acids	2969	C01571
Octanoic acid	HMDB0000482	Organic compounds	Lipids and lipid-like molecules	Fatty Acyls	Fatty acids and conjugates	Medium-chain fatty acids	379	C06423
Chenodeoxycholic acid	HMDB0000518	Organic compounds	Lipids and lipid-like molecules	Steroids and steroid derivatives	Bile acids, alcohols and derivatives	Dihydroxy bile acids, alcohols and derivatives	10133	C02528
Cinnamic acid	HMDB0000567	Organic compounds	Phenylpropanoids and polyketides	Cinnamic acids and derivatives	Cinnamic acids	Cinnamic acids	5372954	C10438
cis_Aconitic acid	HMDB0000072	Organic compounds	Organic acids and derivatives	Carboxylic acids and derivatives	Tricarboxylic acids and derivatives	Tricarboxylic acids and derivatives	643757	C00417
Citraconic acid	HMDB0000634	Organic compounds	Lipids and lipid-like molecules	Fatty Acyls	Fatty acids and conjugates	Methyl-branched fatty acids	643798	C02226
Citramalic acid	HMDB0000426	Organic compounds	Lipids and lipid-like molecules	Fatty Acyls	Fatty acids and conjugates	Hydroxy fatty acids	1081	C00815
Citric acid	HMDB0000094	Organic compounds	Organic acids and derivatives	Carboxylic acids and derivatives	Tricarboxylic acids and derivatives	Tricarboxylic acids and derivatives	311	C00158
Citrulline	HMDB000	Organic c	Organic acids and	Fatty Acyls	Amino acids,	L-alpha-amino	9750	C00327

	0904	compounds	derivatives		peptides, and analogues	acids		
Creatine	HMDB0000064	Organic compounds	Organic acids and derivatives	Fatty Acyls	Amino acids, peptides, and analogues	Alpha amino acids and derivatives	586	C00300
Deoxycholic acid	HMDB0000626	Organic compounds	Lipids and lipid-like molecules	Steroids and steroid derivatives	Bile acids, alcohols and derivatives	Dihydroxy bile acids, alcohols and derivatives	222528	C04483
Docosahexaenoic acid DHA	HMDB0002183	Organic compounds	Lipids and lipid-like molecules	Fatty Acyls	Fatty acids and conjugates	Very long-chain fatty acids	445580	C06429
Dihomo-gamma-Linolenic Acid	HMDB0002925	Organic compounds	Lipids and lipid-like molecules	Fatty Acyls	Fatty acids and conjugates	Long-chain fatty acids	5280581	C03242
Dimethylglycine	HMDB0000092	Organic compounds	Organic acids and derivatives	Fatty Acyls	Amino acids, peptides, and analogues	Alpha amino acids	673	C01026
Dodecanoic acid	HMDB0000638	Organic compounds	Lipids and lipid-like molecules	Fatty Acyls	Fatty acids and conjugates	Medium-chain fatty acids	3893	C02679
Docosapentaenoic acid DPA	HMDB0006528	Organic compounds	Lipids and lipid-like molecules	Fatty Acyls	Fatty acids and conjugates	Very long-chain fatty acids	5497182	C16513
Docosapentaenoic acid 22n_6	HMDB0001976	Organic compounds	Lipids and lipid-like molecules	Fatty Acyls	Fatty acids and conjugates	Very long-chain fatty acids	6441454	C16513
Eicosapentaenoic acid EPA	HMDB0001999	Organic compo	Lipids and lipid-like molecules	Fatty Acyls	Fatty acids and conjugates	Long-chain fatty acids	446284	C06428

		unds						
D_Fructose	HMDB0000660	Organic compounds	Organic oxygen compounds	Organooxygen compounds	Carbohydrates and carbohydrate conjugates	C-glycosyl compounds	439709	C02336
Fumaric acid	HMDB0000134	Organic compounds	Organic acids and derivatives	Carboxylic acids and derivatives	Dicarboxylic acids and derivatives	Dicarboxylic acids and derivatives	444972	C00122
Gamma_Linolenic acid	HMDB0003073	Organic compounds	Lipids and lipid-like molecules	Fatty Acyls	Lineolic acids and derivatives	Lineolic acids and derivatives	5280933	C06426
Glycocholic acid	HMDB0000138	Organic compounds	Lipids and lipid-like molecules	Steroids and steroid derivatives	Bile acids, alcohols and derivatives	Glycinated bile acids and derivatives	10140	C01921
Glycochenodeoxycholic acid	HMDB0000637	Organic compounds	Lipids and lipid-like molecules	Steroids and steroid derivatives	Bile acids, alcohols and derivatives	Glycinated bile acids and derivatives	12544	C05466
Glycodeoxycholic acid	HMDB0000631	Organic compounds	Lipids and lipid-like molecules	Steroids and steroid derivatives	Bile acids, alcohols and derivatives	Glycinated bile acids and derivatives	22833539	C05464
71 Glycolithocholic acid_3_Sulfate	HMDB0002639	Organic compounds	Lipids and lipid-like molecules	Steroids and steroid derivatives	Bile acids, alcohols and derivatives	Glycinated bile acids and derivatives	72222	C11301
D_Gluconolactone	HMDB0000150	Organic compounds	Organic oxygen compounds	Organooxygen compounds	Carbohydrates and carbohydrate conjugates	Gluconolactones	7027	C00198
D_Glucose	HMDB0000122	Organic compo	Organic oxygen compounds	Organooxygen compounds	Carbohydrates and carbohydrate	Hexoses	64689	C00221

		unds			conjugates			
L_Glutamic acid	HMDB0000148	Organic compounds	Organic acids and derivatives	Carboxylic acids and derivatives	Amino acids, peptides, and analogues	Glutamic acid and derivatives	33032	C00025
Glutaric acid	HMDB0000661	Organic compounds	Organic acids and derivatives	Carboxylic acids and derivatives	Dicarboxylic acids and derivatives	Dicarboxylic acids and derivatives	743	C00489
Glyceric acid	HMDB0000139	Organic compounds	Organic oxygen compounds	Organooxygen compounds	Carbohydrates and carbohydrate conjugates	Sugar acids and derivatives	439194	C00258
Glycine	HMDB0000123	Organic compounds	Organic acids and derivatives	Carboxylic acids and derivatives	Amino acids, peptides, and analogues	Alpha amino acids	750	C00037
Glycolic acid	HMDB0000115	Organic compounds	Organic acids and derivatives	Hydroxy acids and derivatives	Alpha hydroxy acids and derivatives	Alpha hydroxy acids and derivatives	757	C03547
Glycoursodeoxycholic acid	HMDB0000708	Organic compounds	Lipids and lipid-like molecules	Steroids and steroid derivatives	Bile acids, alcohols and derivatives	Glycinated bile acids and derivatives	12310288	
Heptanoic acid	HMDB0000666	Organic compounds	Lipids and lipid-like molecules	Fatty Acyls	Fatty acids and conjugates	Medium-chain fatty acids	8094	C17714
Hippuric acid	HMDB0000714	Organic compounds	Benzenoids	Benzene and substituted derivatives	Benzoic acids and derivatives	Hippuric acids	464	C01586
L_Histidine	HMDB0000177	Organic compounds	Organic acids and derivatives	Carboxylic acids and derivatives	Amino acids, peptides, and analogues	Histidine and derivatives	6274	C00135

Homovanillic acid	HMDB0000118	Organic compounds	Benzenoids	Phenols	Methoxyphenols	Methoxyphenols	1738	C05582
Hydrocinnamic acid	HMDB0000764	Organic compounds	Phenylpropanoids and polyketides	Phenylpropanoic acids	NA	Phenylpropanoic acids	107	C05629
Imidazolepropionic acid	HMDB0002271	Organic compounds	Organoheterocyclic compounds	Azoles	Imidazoles	Imidazolyl carboxylic acids and derivatives	70630	C20522
3_Indolepropionic acid	HMDB0002302	Organic compounds	Organoheterocyclic compounds	Indoles and derivatives	Indolyl carboxylic acids and derivatives	Indolyl carboxylic acids and derivatives	3744	NA
Indoleacetic acid	HMDB0000197	Organic compounds	Organoheterocyclic compounds	Indoles and derivatives	Indolyl carboxylic acids and derivatives	Indole-3-acetic acid derivatives	802	C00954
Indoleacetic acid	HMDB0000197	Organic compounds	Organoheterocyclic compounds	Indoles and derivatives	Indolyl carboxylic acids and derivatives	Indole-3-acetic acid derivatives	802	C00954
Isobutyric acid	HMDB0001873	Organic compounds	Organic acids and derivatives	Carboxylic acids and derivatives	Carboxylic acids	Carboxylic acids	6590	C02632
Isocitric acid	HMDB0000193	Organic compounds	Organic acids and derivatives	Carboxylic acids and derivatives	Tricarboxylic acids and derivatives	Tricarboxylic acids and derivatives	1198	C00311
L_Isoleucine	HMDB0000172	Organic compounds	Organic acids and derivatives	Carboxylic acids and derivatives	Amino acids, peptides, and analogues	Isoleucine and derivatives	6306	C00407
Isovaleric acid	HMDB000	Organic compo	Lipids and lipid-like	Fatty Acyls	Fatty acids and	Methyl-branched	10430	C08262

	0718	unds	molecules		conjugates	fatty acids		
Ketoleucine	HMDB0000695	Organic compounds	Organic acids and derivatives	Keto acids and derivatives	Short-chain keto acids and derivatives	Short-chain keto acids and derivatives	70	C00233
L_Kynurenine	HMDB0000684	Organic compounds	Organic oxygen compounds	Organooxygen compounds	Carbonyl compounds	Alkyl-phenylketones	161166	C00328
L_Glutamine	HMDB0000641	Organic compounds	Organic acids and derivatives	Carboxylic acids and derivatives	Amino acids, peptides, and analogues	L-alpha-amino acids	5961	C00064
L_Lactic acid	HMDB0000190	Organic compounds	Organic acids and derivatives	Hydroxy acids and derivatives	Alpha hydroxy acids and derivatives	Alpha hydroxy acids and derivatives	107689	C00186
Carnitine	HMDB0000062	Organic compounds	Organic nitrogen compounds	Organonitrogen compounds	Quaternary ammonium salts	Carnitines	10917	C00318
L_Leucine	HMDB0000687	Organic compounds	Organic acids and derivatives	Carboxylic acids and derivatives	Amino acids, peptides, and analogues	Leucine and derivatives	6106	C00123
Linoleic acid	HMDB0000673	Organic compounds	Lipids and lipid-like molecules	Fatty Acyls	Lineolic acids and derivatives	Lineolic acids and derivatives	5280450	C01595
L_Lysine	HMDB0000182	Organic compounds	Organic acids and derivatives	Carboxylic acids and derivatives	Amino acids, peptides, and analogues	L-alpha-amino acids	5962	C00047
L_Malic acid	HMDB0000156	Organic compounds	Organic acids and derivatives	Hydroxy acids and derivatives	Beta hydroxy acids and derivatives	Beta hydroxy acids and derivatives	222656	C00149

Malonic acid	HMDB0000691	Organic compounds	Organic acids and derivatives	Carboxylic acids and derivatives	Dicarboxylic acids and derivatives	Dicarboxylic acids and derivatives	867	C04025
L_Methionine	HMDB0000696	Organic compounds	Organic acids and derivatives	Carboxylic acids and derivatives	Amino acids, peptides, and analogues	Methionine and derivatives	6137	C00073
Methylcysteine	HMDB0002108	Organic compounds	Organic acids and derivatives	Carboxylic acids and derivatives	Amino acids, peptides, and analogues	Cysteine and derivatives	24417	NA
Methylmalonic acid	HMDB0000202	Organic compounds	Organic acids and derivatives	Carboxylic acids and derivatives	Dicarboxylic acids and derivatives	Dicarboxylic acids and derivatives	487	C02170
Myristic acid	HMDB0000806	Organic compounds	Lipids and lipid-like molecules	Fatty Acyls	Fatty acids and conjugates	Long-chain fatty acids	11005	C06424
Myristoleic acid	HMDB0002000	Organic compounds	Lipids and lipid-like molecules	Fatty Acyls	Fatty acids and conjugates	Long-chain fatty acids	5281119	C08322
N_Phenylacetylphenylalanine	HMDB0002372	Organic compounds	Organic acids and derivatives	Carboxylic acids and derivatives	Amino acids, peptides, and analogues	Phenylalanine and derivatives	47579	NA
Oleic acid	HMDB0000207	Organic compounds	Lipids and lipid-like molecules	Fatty Acyls	Fatty acids and conjugates	Long-chain fatty acids	445639	C00712
Ornithine	HMDB0000214	Organic compounds	Organic acids and derivatives	Carboxylic acids and derivatives	Amino acids, peptides, and analogues	L-alpha-amino acids	6262	C00077

Oxalic acid	HMDB0002329	Organic compounds	Organic acids and derivatives	Carboxylic acids and derivatives	Dicarboxylic acids and derivatives	Dicarboxylic acids and derivatives	971	C00209
Oxoadipic acid	HMDB0000225	Organic compounds	Organic acids and derivatives	Keto acids and derivatives	Medium-chain keto acids and derivatives	Medium-chain keto acids and derivatives	71	C00322
Oxoglutaric acid	HMDB0000208	Organic compounds	Organic acids and derivatives	Keto acids and derivatives	Gamma-keto acids and derivatives	Gamma-keto acids and derivatives	51	C00026
Palmitoleic acid	HMDB0003229	Organic compounds	Lipids and lipid-like molecules	Fatty Acyls	Fatty acids and conjugates	Long-chain fatty acids	445638	C08362
Pelargonic acid	HMDB00847	Organic compounds	Lipids and lipid-like molecules	Fatty Acyls	Fatty acids and conjugates	Medium-chain fatty acids	8158	C01601
Pentadecanoic acid	HMDB0000826	Organic compounds	Lipids and lipid-like molecules	Fatty Acyls	Fatty acids and conjugates	Long-chain fatty acids	13849	C16537
Phenylacetylglutamine	HMDB0006344	Organic compounds	Organic acids and derivatives	Carboxylic acids and derivatives	Amino acids, peptides, and analogues	N-acyl-alpha amino acids	92258	C04148
L_Phenylalanine	HMDB0000159	Organic compounds	Organic acids and derivatives	Carboxylic acids and derivatives	Amino acids, peptides, and analogues	Phenylalanine and derivatives	6140	C00079
Phenyllactic acid	HMDB0000779	Organic compounds	Benzenoids	Benzene and substituted derivatives	NA	Benzene and substituted derivatives	1303	NA
Phenylpyruvic acid	HMDB000	Organic compo	Benzenoids	Benzene and substituted	Phenylpyruvic acid	Phenylpyruvic acid	997	C00166

	0205	unds		derivatives	derivatives	derivatives		
Phthalic acid	HMDB0002107	Organic compounds	Benzenoids	Benzene and substituted derivatives	Benzoic acids and derivatives	Benzoic acids	1017	C01606
p_Hydroxyphenylacetic acid	HMDB0000020	Organic compounds	Benzenoids	Phenols	1-hydroxy-2-unsubstituted benzenoids	1-hydroxy-2-unsubstituted benzenoids	127	C00642
L_Proline	HMDB0000162	Organic compounds	Organic acids and derivatives	Carboxylic acids and derivatives	Amino acids, peptides, and analogues	Proline and derivatives	145742	C00148
Propionic acid	HMDB00237	Organic compounds	Organic acids and derivatives	Carboxylic acids and derivatives	Carboxylic acids	Carboxylic acids	1032	C00163
Propionylcarnitine	HMDB0000824	Organic compounds	Lipids and lipid-like molecules	Fatty Acyls	Fatty acid esters	Acyl carnitines	188824	C03017
Pyroglutamic acid	HMDB0000267	Organic compounds	Organic acids and derivatives	Carboxylic acids and derivatives	Amino acids, peptides, and analogues	Alpha amino acids and derivatives	7405	C01879
Rhamnose	HMDB0000849	Organic compounds	Organic oxygen compounds	Organooxygen compounds	Carbohydrates and carbohydrate conjugates	Hexoses	25310	C00507
D_Ribulose	HMDB0000621	Organic compounds	Organic oxygen compounds	Organooxygen compounds	Carbohydrates and carbohydrate conjugates	Pentoses	12358756	C00309

L_Serine	HMDB0000187	Organic compounds	Organic acids and derivatives	Carboxylic acids and derivatives	Amino acids, peptides, and analogues	Serine and derivatives	5951	C00065
Suberic acid	HMDB0000893	Organic compounds	Lipids and lipid-like molecules	Fatty Acyls	Fatty acids and conjugates	Medium-chain fatty acids	10457	C08278
Succinic acid	HMDB0000254	Organic compounds	Organic acids and derivatives	Carboxylic acids and derivatives	Dicarboxylic acids and derivatives	Dicarboxylic acids and derivatives	1738118	C00042
Tartaric acid	HMDB0000956	Organic compounds	Organic oxygen compounds	Organooxygen compounds	Carbohydrates and carbohydrate conjugates	Sugar acids and derivatives	444305	C00898
Taurocholic acid	HMDB0000036	Organic compounds	Lipids and lipid-like molecules	Steroids and steroid derivatives	Bile acids, alcohols and derivatives	Trihydroxy bile acids, alcohols and derivatives	6675	C05122
Taurodeoxycholic acid	HMDB0000896	Organic compounds	Lipids and lipid-like molecules	Steroids and steroid derivatives	Bile acids, alcohols and derivatives	Taurinated bile acids and derivatives	2733768	C05463
Taurohyodeoxycholic acid	NA	NA	NA	NA	NA	NA	119046	NA
Taurochenodeoxycholic acid	HMDB0000951	Organic compounds	Lipids and lipid-like molecules	Steroids and steroid derivatives	Bile acids, alcohols and derivatives	Taurinated bile acids and derivatives	387316	C05465
Threonic acid	HMDB0000943	Organic compounds	Organic acids and derivatives	Organooxygen compounds	Carbohydrates and carbohydrate conjugates	Sugar acids and derivatives	5460407	C01620
L_threonin	HMDB000	Organic	Organic acids and	Carboxylic acids and	Amino acids,	L-alpha-amino	6288	C00188

e	0167	compounds	derivatives	derivatives	peptides, and analogues	acids		
L_Tryptophan	HMDB0000929	Organic compounds	Organoheterocyclic compounds	Indoles and derivatives	Indolyl carboxylic acids and derivatives	Indolyl carboxylic acids and derivatives	6305	C00078
L_Tyrosine	HMDB0000158	Organic compounds	Organic acids and derivatives	Carboxylic acids and derivatives	Amino acids, peptides, and analogues	Tyrosine and derivatives	6057	C00082
Beta_Ursodeoxycholic acid	HMDB0000686	Organic compounds	Lipids and lipid-like molecules	Steroids and steroid derivatives	Bile acids, alcohols and derivatives	Dihydroxy bile acids, alcohols and derivatives	127601	C17662
Valeric acid	HMDB0000892	Organic compounds	Lipids and lipid-like molecules	Fatty Acyls	Fatty acids and conjugates	Straight chain fatty acids	7991	C00803
L_Valine	HMDB0000883	Organic compounds	Organic acids and derivatives	Carboxylic acids and derivatives	Amino acids, peptides, and analogues	Valine and derivatives	6287	C00183
D_Xylose	HMDB0000098	Organic compounds	Organic oxygen compounds	Organooxygen compounds	Carbohydrates and carbohydrate conjugates	Pentoses	135191	C00181
D_Xylulose	HMDB0001644	Organic compounds	Organic oxygen compounds	Organooxygen compounds	Carbohydrates and carbohydrate conjugates	Pentoses	12358757	C00310

Table S3. List of Metabolites Selected by Univariate Analysis

Metabolites	*P*-value	-LOG10(*P*-value)
Isoleucine	0.049667166	1.303930619
2-Hydroxybutyric acid	0.043451987	1.361990363
DPAn-6	0.041611222	1.380789532
Lactic acid	0.03924064	1.406263923
alpha-Linolenic acid	0.026236885	1.581087727
Leucine	0.025727465	1.589602999
Gluconolactone	0.019492888	1.71012381
Valine	0.017161952	1.765433311
Indole-3-propionic acid	0.013212009	1.879031146
Ornithine	0.00707507	2.150269277

Table S4. List of Metabolites Selected by Multivariate Analysis

Metabolites	VIP	*P*（corr.）
alpha-Ketoisovaleric acid	1.00931272053034	-0.1559
Phenyllactic acid	1.02902760510718	-0.15894
Lysine	1.05406624903568	-0.16281
GUDCA	1.05607940632992	0.16312
Ketoleucine	1.07775615363571	-0.16647
Pyroglutamic acid	1.08009986693529	-0.16683
Homovanillic acid	1.09732149621063	-0.16949
3-Methyl-2-oxopentanoic acid	1.10224589377668	-0.17025
Imidazolepropionic acid	1.10937125387202	-0.17135
3-Hydroxybutyric acid	1.1306530669975	-0.17464
Myristic acid	1.14646353820334	0.17708
Acetic acid	1.16205941097146	-0.17949
Proline	1.17567188098116	-0.18159
Phthalic acid	1.19013712654801	0.18383
Phenylalanine	1.19366437239953	-0.18437
p-Hydroxyphenylacetic acid	1.20205647851799	-0.18567
Pelargonic acid	1.22012382456206	0.18846
Arachidonic acid	1.22629196882315	0.18941

Tartaric acid	1.26075089014625	-0.19473
Indolelactic acid	1.28587236201767	-0.19862
Isocitric acid	1.31905408290258	-0.20374
Glucose	1.34669869039791	-0.20801
Myristoleic acid	1.35056660834942	0.20861
Glycolic acid	1.39500903485192	-0.21547
Ribulose	1.43630646072318	-0.22185
CA	1.4510058147264	-0.22412
Azelaic acid	1.49348398131827	0.23068
DPAn-6	1.52014501039281	0.2348
DHA	1.52665764491366	0.23581
Lactic acid	1.57368640245074	-0.24307
3-Aminoisobutanoic acid	1.61235976858652	-0.24904
Histidine	1.73350771881976	-0.26776
CDCA	1.82331506419948	-0.28163
Pentadecanoic acid	1.89457076729554	0.29263
Gluconolactone	1.95202523567371	-0.30151
2-Hydroxybutyric acid	1.98576412132938	-0.30672
Indole-3-propionic acid	2.08230178165829	0.32163
Isoleucine	2.13694617652882	-0.33007
Leucine	2.34889965894143	-0.36281

Ornithine	2.38123365721444	-0.3678
Valine	2.39166206486614	-0.36942
alpha-Linolenic acid	2.3968599969224	-0.37022

Table S5. List of Cytokines

Cytokines	BPN group	LUAD group	*P*-value
IFN-gamma (median [IQR])	0.000 [0.000, 0.178]	0.000 [0.000, 0.000]	0.4909
IL-12p70 (median [IQR])	1.190 [0.300, 2.183]	0.280 [0.000, 2.020]	0.0541
IL-13 (median [IQR])	8.055 [4.060, 12.230]	5.195 [0.000, 11.130]	0.2346
IL-1beta (median [IQR])	2.580 [1.440, 4.145]	1.920 [0.000, 2.580]	0.0727
IL-2 (median [IQR])	14.075 [5.345, 19.430]	4.745 [0.000, 14.157]	0.0583
IL-4 (median [IQR])	4.240 [3.190, 4.740]	3.720 [0.000, 4.740]	0.4216
IL-5 (median [IQR])	3.975 [1.675, 7.142]	1.890 [0.232, 5.805]	0.1027
IL-6 (median [IQR])	0.000 [0.000, 0.000]	0.000 [0.000, 0.000]	0.7216
TNF-alpha (median [IQR])	3.280 [0.000, 3.680]	1.460 [0.000, 2.880]	0.2524
GM-CSF (median [IQR])	19.880 [2.470, 25.460]	11.935 [0.000, 23.732]	0.0961
IL-18 (median [IQR])	21.790 [16.498, 41.105]	21.260 [15.480, 28.320]	0.42
IL-10 (median [IQR])	0.000 [0.000, 0.708]	0.000 [0.000, 0.200]	0.3092
IL-17A (median [IQR])	7.850 [5.210, 16.798]	5.270 [2.220, 9.490]	0.0226
IL-21 (median [IQR])	0.000 [0.000, 2.790]	0.000 [0.000, 3.725]	0.7358
IL-22 (median [IQR])	0.000 [0.000, 6.700]	0.000 [0.000, 5.370]	0.6238
IL.23 (median [IQR])	0.000 [0.000, 0.900]	0.000 [0.000, 0.050]	0.5005
IL-27 (median [IQR])	35.315 [23.770, 52.727]	26.320 [15.020, 38.230]	0.0462
IL-31 (median [IQR])	0.810 [0.000, 1.848]	3.000 [0.045, 4.040]	0.0488
IL-15 (median [IQR])	7.315 [3.703, 12.683]	3.570 [2.130, 7.150]	0.0248

IL-1alpha (median [IQR])	0.130 [0.000, 0.235]	0.130 [0.000, 0.223]	0.5056
IL-1RA (median [IQR])	221.085 [93.870, 345.782]	137.910 [85.190, 225.450]	0.1673
IL-7 (median [IQR])	0.750 [0.390, 1.152]	0.540 [0.035, 1.080]	0.2669
Eotaxin (median [IQR])	11.330 [9.242, 14.265]	11.935 [9.707, 14.750]	0.499
GRO-alpha (median [IQR])	1.335 [0.128, 2.702]	2.260 [0.578, 2.898]	0.4397
IL-8 (median [IQR])	0.060 [0.000, 0.332]	0.365 [0.000, 0.945]	0.0902
IP-10 (median [IQR])	10.295 [6.732, 13.938]	17.625 [12.293, 22.132]	0.0041
MCP-1 (median [IQR])	25.240 [19.038, 31.208]	30.385 [21.445, 40.850]	0.3105
MIP-1alpha (median [IQR])	1.110 [0.732, 1.418]	0.990 [0.778, 1.420]	0.8099
MIP-1beta (median [IQR])	29.650 [24.388, 37.360]	33.350 [27.525, 39.120]	0.3944
SDF-1alpha (median [IQR])	194.535 [140.370, 290.783]	276.555 [215.000, 341.918]	0.0585
